# Arrangement of the *Clostridium baratii* F7 Toxin Gene Cluster with Identification of a σ Factor That Recognizes the Botulinum Toxin Gene Cluster Promoters

**DOI:** 10.1371/journal.pone.0097983

**Published:** 2014-05-22

**Authors:** Nir Dover, Jason R. Barash, Julianne N. Burke, Karen K. Hill, John C. Detter, Stephen S. Arnon

**Affiliations:** 1 Infant Botulism Treatment and Prevention Program, California Department of Public Health, Richmond, California, United States of America; 2 Bioscience Division, Los Alamos National Laboratory, Los Alamos, New Mexico, United States of America; University of Illinois at Chicago College of Medicine, United States of America

## Abstract

Botulinum neurotoxin (BoNT) is the most poisonous substances known and its eight toxin types (A to H) are distinguished by the inability of polyclonal antibodies that neutralize one toxin type to neutralize any of the other seven toxin types. Infant botulism, an intestinal toxemia orphan disease, is the most common form of human botulism in the United States. It results from swallowed spores of *Clostridium botulinum* (or rarely, neurotoxigenic *Clostridium butyricum* or *Clostridium baratii*) that germinate and temporarily colonize the lumen of the large intestine, where, as vegetative cells, they produce botulinum toxin. Botulinum neurotoxin is encoded by the *bont* gene that is part of a toxin gene cluster that includes several accessory genes. We sequenced for the first time the complete botulinum neurotoxin gene cluster of nonproteolytic *C. baratii* type F7. Like the type E and the nonproteolytic type F6 botulinum toxin gene clusters, the *C. baratii* type F7 had an *orfX* toxin gene cluster that lacked the regulatory *botR* gene which is found in proteolytic *C. botulinum* strains and codes for an alternative σ factor. In the absence of *botR*, we identified a putative alternative regulatory gene located upstream of the *C. baratii* type F7 toxin gene cluster. This putative regulatory gene codes for a predicted σ factor that contains DNA-binding-domain homologues to the DNA-binding domains both of BotR and of other members of the TcdR-related group 5 of the σ^70^ family that are involved in the regulation of toxin gene expression in clostridia. We showed that this TcdR-related protein in association with RNA polymerase core enzyme specifically binds to the *C. baratii* type F7 botulinum toxin gene cluster promoters. This TcdR-related protein may therefore be involved in regulating the expression of the genes of the botulinum toxin gene cluster in neurotoxigenic *C. baratii*.

## Introduction

Infant botulism caused by nonproteolytic neurotoxigenic *Clostridium baratii* type F is rare and is notable for its severity and rapidity of onset [1]–[6]. BoNT/F is also produced by proteolytic *Clostridium botulinum* and by nonproteolytic *C. botulinum*. The type F toxins produced by these organisms have been distinguished into seven subtypes with *C. baratii* toxin categorized as subtype F7 [7].

Botulinum neurotoxin is encoded by the *bont* gene that is part of a gene cluster that includes several nontoxin accessory genes. Two main *bont* gene cluster organizations are known; The hemagglutinin (*ha*) toxin gene cluster is found in *C. botulinum* types A1, A5, B, C, D and G strains, while the *orfX* toxin gene cluster is found in *C. botulinum* types A1–A4, E, F and H, *C. butyricum* type E and *C. baratii* type F strains [8]–[11]. The toxin gene clusters of proteolytic *C. botulinum* (toxin types A, B, F and H) contain the regulatory gene *botR* in both the *ha* and *orfX* toxin gene clusters that codes for a σ factor that positively controls expression of the structural gene for botulinum toxin as well as of its accessory genes [9], [12]–[14]. Paradoxically, the *botR* regulatory gene is not present in the toxin gene clusters of nonproteolytic *C. botulinum* type E, nonproteolytic *C. botulinum* type F and nonproteolytic *C. butyricum* type E, yet all of these strains produce botulinum toxin [8], [15], [16].

The structural genes of the *orfX* toxin gene cluster are transcribed as two divergent polycistronic transcripts. One transcript encodes the *orfX1*, *orfX2* and *orfX3* genes, while the second transcript encodes the *p47*, *ntnh* and *bont* genes [17], [18]. A similar divergent polycistronic transcription pattern occurs in the botulinum *ha* toxin gene clusters [19]–[21]. The structural genes are transcribed from conserved promoter sequences located upstream of *orfX1* and *p47* that are recognized and activated by BotR [17], [18], [22], [23].

Bacterial RNA polymerase holoenzyme is a multisubunit protein that consists of a core enzyme and a dissociable σ subunit that is responsible for recognizing DNA promoter sequences [24], [25]. Most RNA transcription in growing bacteria begins with a primary σ factor (σ^70^ in *E. coli* and σ^A^ in Gram-positive bacteria) that associates with the RNA polymerase core enzyme. However, several alternative σ factors can replace the primary σ factor when adaptation to specific stresses or morphological development is required [25], [26]. The most well-characterized DNA promoter sequence elements recognized by σ factors are the −35 and −10 elements (TTGACA and TATAAT, respectively, as the *E. coli* σ^70^ consensus recognition sequences), which are designated by their approximate nucleotide distance from the transcription start site.

The botulinum toxin regulatory protein BotR is a σ factor in the TcdR-related group 5 within the σ^70^ family of σ factors. This σ^70^ family group 5 includes regulators of several clostridial toxins; in *Clostridium difficile* TcdR regulates expression of the difficile toxins A and B genes (*tcdA* and *tcdB*), in *C. tetani* TetR regulates expression of the tetanus toxin gene (*tetX*) and in *C. perfringens* UviA regulates expression of a gene (*bcn*) that codes for its bacteriocin BCN5 [14], [25], [27].

We determined by sequencing the complete organization of the botulinum toxin gene cluster of nonproteolytic neurotoxigenic *C. baratii* type F7, in order to better understand its mechanism(s) of toxin production and to ascertain whether it might contain a *botR* gene. We found that the bacterium contained an *orfX* toxin gene cluster that lacked the regulatory *botR* gene. Unexpectedly, immediately upstream of the type F7 toxin gene cluster, we found a two-gene operon that resembled the *C. perfringens uviAB* operon. We further found that *C. baratii* type F7 contains BotR-recognized conserved DNA sequences thar are found in all *C. botulinum* strains that carry *botR*. We showed that the *C. baratii* type F7 UviA-like protein, in complex with RNA polymerase core enzyme, specifically recognizes and binds to the *C. baratii* type F7 botulinum toxin gene cluster promoters. This UviA-like, TcdR-related σ factor of neurotoxigenic *C. baratii* type F7 may participate in regulating production of its botulinum neurotoxin.

## Materials and Methods

### Ethics statement

All animal work was conducted in accord with the standards of the Association for the Assessment and Accreditation of Laboratory Animal Care (AAALAC) and was approved by the California Department of Public Health (CDPH) Institutional Animal Care and Use Committee (IACUC) under Animal Use Protocol #12-02.

### Bacterial strains and culture conditions

The neurotoxigenic *Clostridium baratii* type F7 strain IBCA03-0045 described in this study was isolated from the feces of a California infant botulism patient [6]. Pure cultures grown from isolated single colonies were cultured in CMGS broth (0.5% yeast extract, 1.8% Criterion extract broth, 0.5% glucose and 0.2% soluble starch) and stored at −75°C in 1% skim milk. The nontoxigenic *C. baratii* strain IBCA08-0076 was isolated from a fecal specimen submitted for infant botulism testing and was determined to be *C*. *baratii* based on colony characteristics, Gram stain morphology, an API 20a biochemical profile (Biomerieux, Hazelwood, MO) and 16S rRNA sequencing (GenBank accession number JX847739). *Clostridium botulinum* type E7 strain Detroit was isolated from a Detroit, MI, foodborne botulism outbreak. *Clostridium butyricum* type E4 strain 109 was isolated from an Italian botulism patient [28]. *Clostridium botulinum* type F6 strain IBCA66-5436 was isolated from a California foodborne botulism outbreak [8], [29]. Botulinum toxin types were determined using the mouse protection assay [30]. A small amount of frozen (−75°C) culture (containing a mixture of CMGS broth and 1% skim milk) was streaked onto 4% Egg Yolk Agar plates (50% Difco egg in brain heart infusion agar) and incubated anaerobically at 35°C for 48 hours. Individual bacterial colonies were removed from the plates, inoculated into 20 ml of pre-reduced TPGY broth (2.5% trypticase peptone, 0.25% protease peptone, 0.2% dextrose, 1% yeast extract, and 0.05% sodium thioglycollate) anaerobically for 24–48 hours at 35°C and then harvested by centrifugation at 3,450 g. The cell pellets were stored at −75°C.

### DNA extraction

Genomic DNA for Sanger sequencing was extracted using the MagNA Pure Compact Nucleic Acid Isolation Kit I and the Bacteria Purification Protocol (Roche Applied Science) according to the manufacturer's instructions. Genomic DNA for next-generation sequencing was extracted using the Qiagen DNeasy Blood and Tissue Kit according to the protocol for Gram-positive bacteria.

### RNA extraction

The bacteria were grown as described above until early stationary phase (OD_600_∼1.3), at which point RNA was extracted with the RNeasy Protect Bacteria Mini Kit (Qiagen) that included treatment with DNase.

### Rapid Amplification of 5' Complementary DNA Ends (5' RACE)

The transcription start sites of the *bont* gene cluster mRNA transcripts were mapped with the 5′ RACE System for Rapid Amplification of cDNA Ends, Version 2.0 (Invitrogen). All primers are listed in Table S1. Primers labeled GSP1 were used for first strand cDNA synthesis; primers labeled GSP2 were used for PCR amplification of the cDNA; primers labeled GSP3 were used for nested PCR amplification and primers labeled seq were used for sequencing of the nested PCR amplicon.

### DNA sequencing

A high-quality draft genome sequence of strain IBCA03-0045 was generated from standard and paired-end libraries that were sequenced on both Illumina and 454 Titanium (Roche Diagnostics) platforms. The 454 data were assembled using Newbler version 2.3, and the Illumina data were assembled with VELVET version 0.7.63. The two assembly results were integrated using parallel Phrap version SPS 4.24 and the integrated assembly was examined with Consed. A review and BLAST search of the draft genome of *C. baratii* strain IBCA03-0045 identified a contig that contained the *bont/F7* gene within an *orfX* botulinum toxin gene cluster. PCR and sequencing primers were designed based on the draft genome sequence (1). PCR was performed with Phusion Hot Start High-Fidelity DNA Polymerase (New England BioLabs, Ipswich, MA) with thermocycling conditions of 98°C for 1 minute and 32 cycles of: 98°C for 20 seconds, 57°C–61°C for 20 seconds and 72°C for 0.5 to 2.5 minutes. Overlapping PCR amplicons were sequenced using an Applied Biosystems 3730XL DNA Analyzer. Primers used for sequencing of *bont/F7* were previously reported by Raphael et al [7]. Primers for sequencing the type F7 botulinum toxin gene cluster, excluding the *bont/F7* gene, were designed during this study. Universal Primers for sequencing of *C. baratii* 16S rRNA were taken from http://en.wikipedia.org/wiki/16S_ribosomal_RNA. For the Sanger sequencing of the regulatory sequences (*orfX1*-*p47* intergenic region) of *C. botulinum* type E7 strain Detroit and *C. butyricum* type E4 strain 109 (GeneBank accession numbers KJ659889 and KJ659890, respectively) genomic DNA was amplified with primers EorfX-GSP2 and Ep47-GSP2 and sequenced with the amplification primers, as well as with primers EorfX-seq and Ep47-GSP3. All primers are listed in Table S1. Sanger DNA sequences were assembled with Sequencher software (Gene Codes, Ann Arbor, MI).

### Sequence analysis and alignments

CLUSTALW multiple alignments of nucleotide sequences, pairwise identities computation and phylogenetic analyses were done with the MEGA4 software [31]. Alignments and comparisons of amino acid sequences were done with the EMBOSS pairwise sequence alignment algorithm and BoxShade (http://www.ch.embnet.org/software/BOX_form.html).

### Protein expression and purification

The complete coding sequence of the *uviA*-like gene of *C. baratii* type F7 strain IBCA03-0045, optimized for *E. coli* expression using GeneGPS algorithm (GeBank accession number KJ659891), was synthetized (DNA2.0) and cloned into a pET-19b expression vector (Novagen) with a 10 histidine-tag at its N-terminus. The plasmid was transformed into *E. coli* strain BL21DE3 (New England BioLabs), and a single colony was picked and grown overnight in Luria-Bertani medium containing 100 µg/ml ampicillin at 37°C. The overnight culture was diluted 1∶100 and isopropyl β-D-1-thiogalactopyranoside (IPTG) (1 mM) was added at an OD_600_ of 1.2 to induce the expression of the target protein. The cells were harvested 6 hours post-IPTG induction. The cell pellet from a 500 ml culture was resuspended in 20 ml of binding buffer (20 mM sodium phosphate, 500 mM NaCl, 20 mM imidazole, pH 7.4) and sonicated for 30 seconds on ice. The lysate was centrifuged at 10,322 g for 20 minutes to remove the supernatant. Inclusion bodies were collected and lysed in 10 ml binding buffer containing 6 M Guanidine-HCl by rocking at room temperature for 30 to 60 min until all pellets were dissolved. The lysate was centrifuged at 10,322 g for 20 min and supernatant was collected. A Ni-charged resin column (GenScript) was first equilibrated with 10 volumes binding buffer containing 6 M Guanidine-HCl and the supernatant was loaded on the column. After washing twice with 10 volumes of binding buffer, the protein was eluted with elution buffer (20 mM sodium phosphate, 500 mM NaCl, 500 mM imidazole, pH 7.4, 6 M Guanidine-HCl) and the eluant was collected. The denatured protein was exchanged into refolding buffer (Virovek) and then exchanged to the final buffer (50 mM Tris-HCl, pH 8.5, 150 mM NaCl, 2% Sarkosyl, 10 mM 2-mercaptoethanol).

### Gel mobility shift assay

Two DNA fragments of 250 bp each corresponding to positions 5909–6158 and 6054–6303 (GenBank accession number JX847735) (−166 bp to +84 bp from the *orfX1* and *p47* transcription start sites, respectively) were synthesized, labeled with biotin at the 5′ terminal and gel purified. Two additional DNA fragments of 250 bp each covering the same positions as above were synthesized but with a modification of positions 5947–6037 and 6176–6235 to a repetitive sequence of ACTG nucleotides. The modified positions correspond to the predicted botulinum toxin gene cluster promoters. *Clostridium baratii* UviA-like protein and *E. coli* RNA polymerase core enzyme (Epicentre) were purified by dialysis and dissolved in phosphate buffered saline (PBS) buffer. 1 µl of labeled DNA (0.1 nM), with or without unlabeled DNA (2 nM), was incubated for 30 minutes with either: 1 µl *C. baratii* UviA-like protein (1.5 µM), 1 µl *E. coli* RNA polymerase core enzyme (300 nM), 2 µl pre-incubated (for 30 minutes) complex of both proteins, or with no added proteins. The incubation reactions at room temperature also contained 2 µl binding buffer (Signosis), 1 µl poly d(I-C) and nuclease-free ddH2O in a total volume of 10 µl. The reactions were loaded on a 4.5% native polyacrylamide gel prepared in Tris-borate-EDTA (TBE) buffer and electrophoresed for 60 minutes. The gels were transferred to a nylon membrane and the probes were immobilized with UV cross-linking. The signals in the blots were detected and analyzed using Signosis EMSA assay kit according to the manufacturer's instructions.

## Results

### Characterization of genes of the type F7 botulinum toxin gene cluster

**Figure 1:**
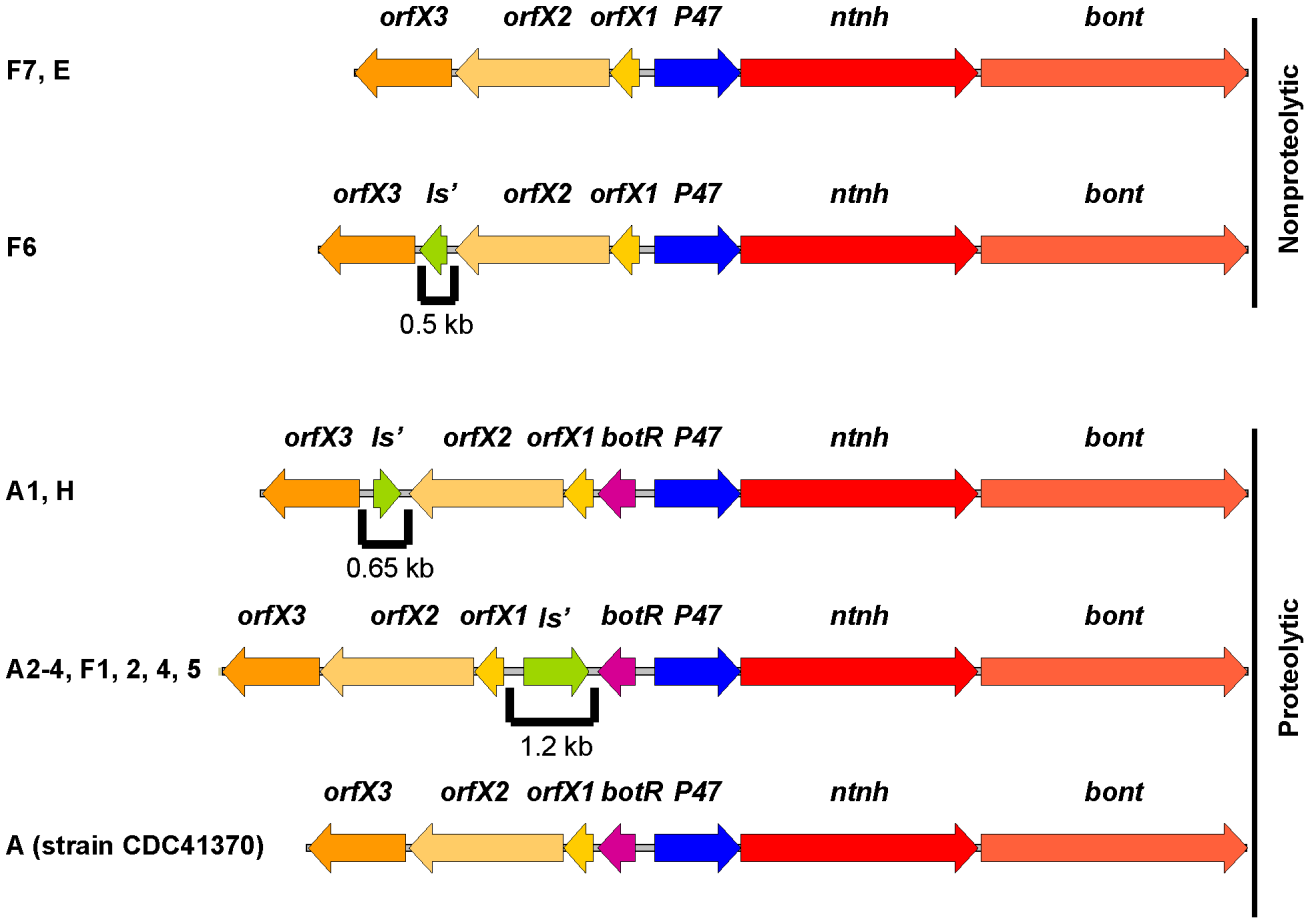
Gene arrangement of the botulinum *orfX* neurotoxin gene clusters in proteolytic and nonproteolytic clostridia. The *C. baratii* F7 toxin gene cluster content and arrangement are identical to those of the type E clusters but are different from those of the F1 and F6 toxin gene clusters. Specifically, the nonproteolytic F7, F6 and E toxin gene clusters lack the *botR* regulatory gene that is present in the proteolytic A, F and H *orfX* toxin gene clusters. The nonproteolytic F6 *orfX2-orfX3* intergenic spacing is not homologous to the proteolytic A1 and H *orfX2-orfX3* intergenic spacing, although all contain a degenerated *is* element. All currently known type E clusters have an identical gene arrangement. The complete neurotoxin gene cluster sequences of type F3 is not available in GenBank and therefore could not be included in this Figure.

The nonproteolytic *C. baratii bont/F7* gene was contained in an *orfX* botulinum toxin gene cluster (GenBank accession number JX847735) (Fig. 1), as has been found in other type F toxin gene clusters [7]. Also, the type F7 toxin gene cluster lacked a *botR* regulatory gene, as is the case with the nonproteolytic *C. botulinum* type E, nonproteolytic *C. butyricum* type E and nonproteolytic *C. botulinum* type F6 toxin gene clusters [8], [16], [32].

Each gene sequence was aligned (CLUSTALW) with its homologs from the *orfX* toxin gene clusters in *C. botulinum* type A, E, and F strains, and the pairwise identities were computed. Most genes of the type F7 cluster shared a low percentage identity with their *orfX* toxin gene clusters homologs in the type A, E, and F strains (Table 1). The pairwise identities of the *bont* genes ranged from 61.0% to 83.7% for nucleotides and from 42.2% to 75.3% for amino acids. The pairwise identities of the non-*bont* genes ranged from 64.2% to 85.4% for nucleotides and from 50.2% to 80.3% for amino acids. Interestingly, the genes of the type F7 toxin gene cluster generally are as similar to their homologs that reside in the proteolytic strains as they are to their homologs that reside in the nonproteolytic strains. Moreover, although both the *C. baratii* type F7 and the *C. botulinum* type F6 toxin gene clusters are nonproteolytic type F clusters that lack the *botR* gene, the *orfX3*, *orfX2*, *orfX1* and *ntnh* genes of the type F7 cluster were the least similar to their type F6 homologs (Table 1).

**Table 1:** Comparison of nucleotide and amino acid identities of the genes of the *C. baratii* type F7 *orfX* cluster to the genes of the *C. botulinum* types A, E, and F *orfX* clusters.

			% nucleotide identity (% amino acid identity)					
Strain	GenBank Accession no.	*bont* Subtype	*orfX3*	*orfX2*	*orfX1*	*p47*	*ntnh*	*bont*
NCTC2916 A(B)	Y14238, AY497357, X52066	A1	82.9 (77.4)	65.8 (50.3)	77.1 (64.3)	78.8 (71.8	84.0 (76.6)	61.8 (42.7)
Kyoto-F	CP001581	A2	82.3 (76.3)	66.1 (50.6)	78.3 (67.1)	80.0 (71.8)	84.0 (76.0)	61.1 (42.2)
CDC41370 Ab	FJ981696	A	82.3 (76.5)	65.7 (50.2)	77.1 (65.0)	80.5 (72.0)	84.0 (76.2)	61.6 (42.7)
Loch Maree	CP000963	A3	82.2 (75.6)	65.6 (50.5)	77.8 (65.7)	80.4 (72.0)	83.9 (75.9)	61.0 (42.9)
657Ba	CP001081	A4	82.2 (76.1)	64.6 (50.9)	79.2 (65.7)	84.2 (77.6)	83.3 (75.5)	61.0 (42.9)
Alaska E43	CP001078	E3	85.4 (80.3)	76.0 (65.6)	74.7 (61.4)	78.4 (67.4)	84.4 (75.6)	76.4 (65.1)
Langeland	CP000728	F1	82.8 (76.7)	66.0 (50.7)	76.1 (62.9)	78.7 (71.5)	84.0 (75.6)	83.7 (75.3)
Bf	ABDP01000023	F2	82.3 (76.0)	66.1 (50.8)	78.4 (65.2)	84.2 (77.4)	84.6 (76.6)	80.7 (70.5)
CDC54086	GU213218	F3	NA	NA	NA	NA	NA	80.8 (70.9)
Af84	AOSX01000018	F4	83.9 (77.8)	82.7 (76.1)	77.7 (65.2)	80.7 (72.7)	83.9 (73.9)	82.7 (73.7)
Af84	AOSX01000021	F5	82.3 (75.6)	66.2 (50.8)	78.4 (65.2)	80.0 (71.8)	84.7 (74.7)	76.4 (65.5)
IBCA66-5436	HQ441176	F6	79.5 (73.8)	64.2 (50.3)	74.5 (62.1)	83.3 (75.4)	79.5 (69.8)	81.5 (71.9)

NA, not available in GenBank.

Note that even the most similar homologous genes were approximately 15% different (nucleotides) and 25% different (amino acids). Also, the most dissimilar homologous genes were approximately 60%–80% different (nucleotides) and 43%–75% different (amino acids). Only one representative of the several known *bont/E* gene clusters (*bont/E3* of strain Alaska E43) was included in Table 1 because the *bont/E* subtype gene cluster sequences are conserved. Both Alaska E43 and IBCA66-5463 are nonproteolytic (Group II), while the other strains in the Table are proteolytic (Group I).

### Analysis of promoters within the type F7 toxin gene cluster

**Figure 2:**
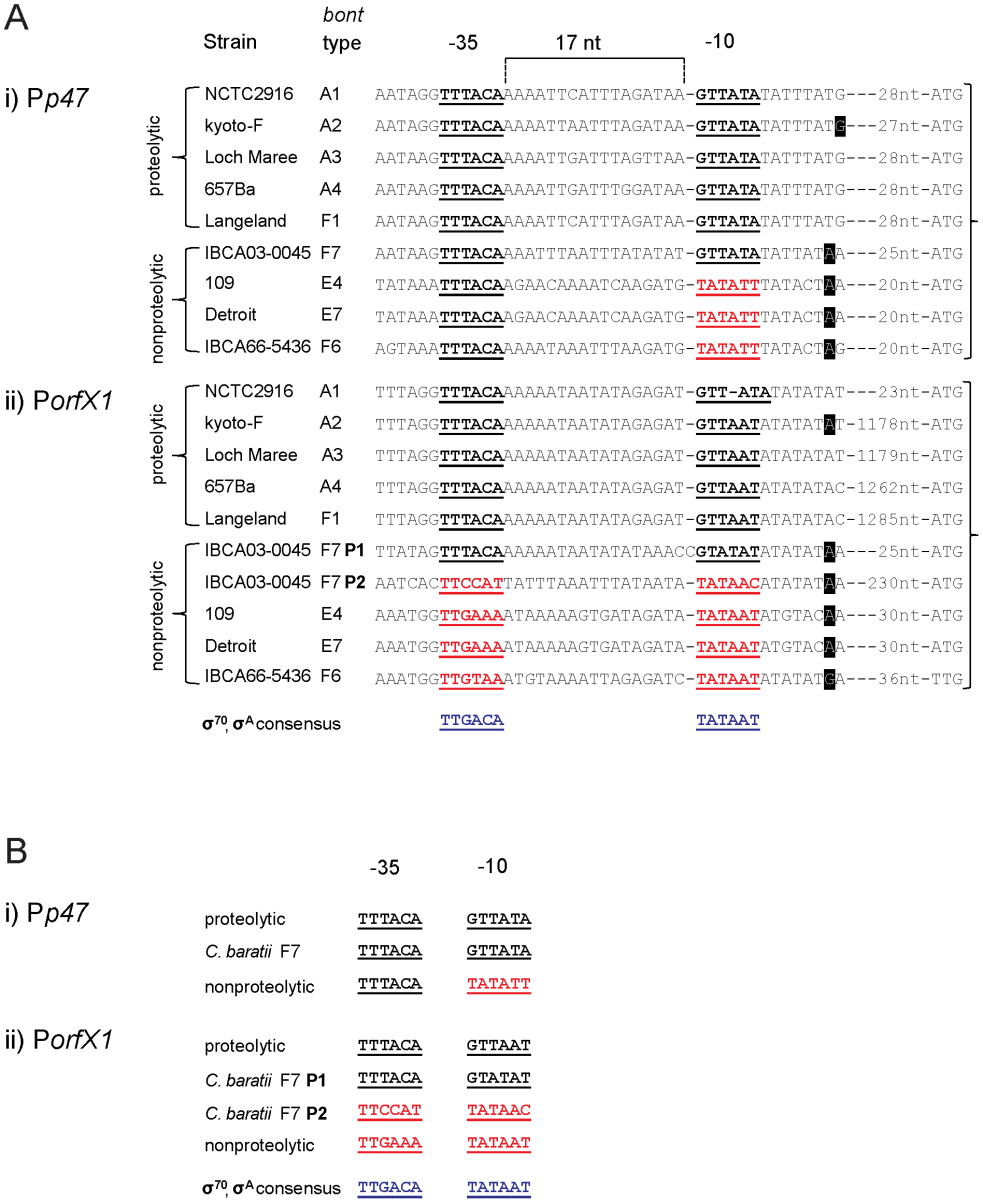
Promoter sequences of *orfX* botulinum toxin gene clusters. A) Aligned sequences of the (i) P*p47* and (ii) P*orfX1* promoters in botulinum *orfX* toxin gene clusters. B) Comparison of the consensus −10 and −35 promoter elements in botulinum *orfX* toxin gene clusters. The sequences of the −10 and −35 promoter elements are bolded and underlined. The BotR-recognized conserved neurotoxin gene cluster promoter elements are bolded and in black. The unique promoter elements of *orfX* toxin gene clusters that reside in nonproteolytic strains are bolded and in red. The σ^70^ and σ^A^ consensus recognition sequences are bolded and in blue. Transcription start sites, where data are available, are blocked in black. Note that the *C. baratii* type F7 P*p47* and P1*orfX1* promoters both contain the conserved, and BotR-recognized, −10 and −35 elements (or a similar sequence). The P*orfX1* −10 and −35 promoter elements in types E and F6 are similar to the consensus sequences recognized by *E. coli* σ^70^ and *Bacillus subtilis* (σ^A^) (bottom row of Figure). F7 P1 and F7 P2 refer to the two putative promoters of the *orfX* operon in *C. baratii* type F7. The two type E strains listed in the Figure represent the two known bacterial species that express type E botulinum toxin, *C. botulinum* (E7) and neurotoxigenic *C. butyricum* (E4).

**Figure 3:**
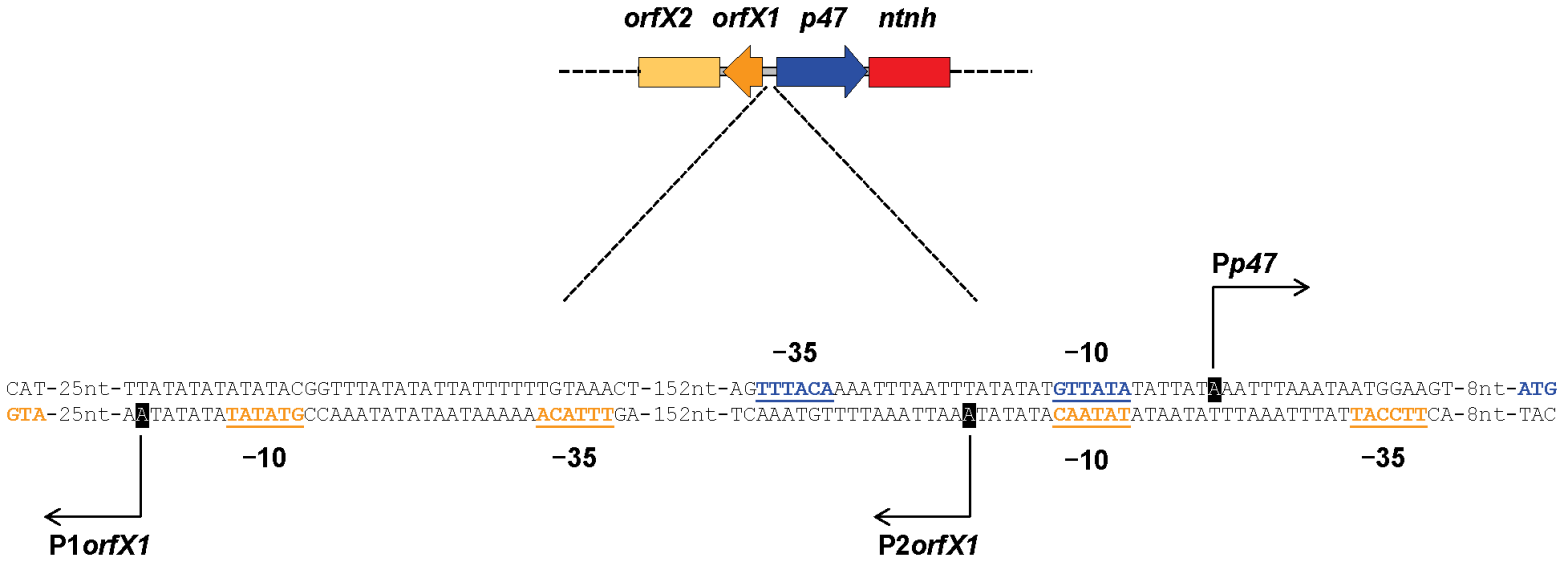
The *orfX1*-*p47* intergenic region containing the *p47* and *orfX1* promoters in *C. baratii* type F7. The *orfX1*-*p47* intergenic region is magnified. The −10 and −35 promoter elements are underlined and colored according to their gene affiliation (*p47* in blue and *orfX1* in gold). The transcription start sites are blocked in black. The translation start codons (ATG) are indicated and colored. Note that the P*p47* and P2*orfX1* promoters share the same −10 element on complementary DNA strands.

Because the regulatory *botR* gene is not present in the botulinum toxin gene clusters of nonproteolytic neurotoxigenic clostridia (Fig. 1), we characterized the promoter sequences of their polycistronic *p47-ntnh-bont* and *orfX1-orfX2-orfX3* transcripts (P*p47* and P*orfX1* respectively) to search for promoter sequences that might be recognized by a presently unknown regulatory protein(s). The transcription start sites of the *p47-ntnh-bont* and the *orfX1-orfX2-orfX3* transcripts in *C. baratii* type F7 strain IBCA03-0045, in *C. botulinum* type F6 strain IBCA66-5436, in *C. botulinum* type E7 strain Detroit and in *C. butyricum* type E4 strain 109 were found using 5′ RACE promoter mapping, and their predicted −10 and −35 promoter elements were identified (Fig. 2). Unexpectedly, in *C. baratii* type F7 two transcription start sites upstream of *orfX1* were identified. One transcription start site, designated P1*orfX1*, was located 27 bp upstream of the transcript start codon, while the second transcription start site, designated P2*orfX1*, was located 232 bp upstream of the transcript start codon (Figs. 2A and 3). Figure 2 compares the aligned *orfX* neurotoxin gene cluster promoter sequences in neurotoxigenic clostridia (both proteolytic and nonproteolytic); the predicted −10 and −35 promoter elements are in bold and underlined [17], [22].

Sequence analysis of the predicted promoters in all *orfX* toxin gene clusters found that the highly conserved −35 element TTTACA, which is recognized by TcdR-related regulatory proteins, was also present in the P*p47* promoter of both proteolytic and nonproteolytic strains (Fig. 2, i). In contrast, the conserved P*p47* −10 element GTTATA was present only in proteolytic *C. botulinum* strains and in nonproteolytic neurotoxigenic *C. baratii*, while the −10 element in the nonproteolytic type E and F6 toxin gene clusters consisted of a TATATT sequence. The −35 element TTTACA recognized by TcdR-related regulatory proteins was also present in the P*orfX1* promoters in proteolytic *C. botulinum* strains and in the *C. baratii* F7 P1*orfX1* promoter. In contrast, the nucleotide sequence of the −35 element of P*orfX1* varied in the nonproteolytic type E, F6 and F7 P2 promoters (TTGAAA, TTGTAA and TTCCAT, respectively) (Fig. 2, ii). The P*orfX1* −10 promoter element GTTAAT was conserved in almost all proteolytic strains but varied in the nonproteolytic strains. Interestingly, the predicted −10 promoter element TATAAT of P*orfX1* in *C. botulinum* types E and F6 and in *C. butyricum* type E is identical to the consensus promoter recognition sequence of the primary (housekeeping) σ factors of *E. coli* (σ^70^) and *Bacillus subtilis* (σ^A^) [33], [34]. This unexpected finding suggests that a regulatory protein closely related to σ^70^ may participate in regulating P*orfX1* in the type E and type F6 toxin gene clusters. The *C. baratii* P1*orfX1* −10 promoter element GTATAT was similar to the P*orfX1* −10 promoter element GTTAAT in proteolytic *C. botulinum* strains, while its P2*orfX1* −10 promoter element TATAAC was similar to the P*orfX1* −10 promoter element TATAAT in the nonproteolytic strains (Fig. 2, ii). Interestingly, in *C. baratii* type F7 the P*p47* promoter and P2*orfX1* promoter share the same predicted −10 element on complementary DNA strands (Fig. 3).

**Figure 4:**
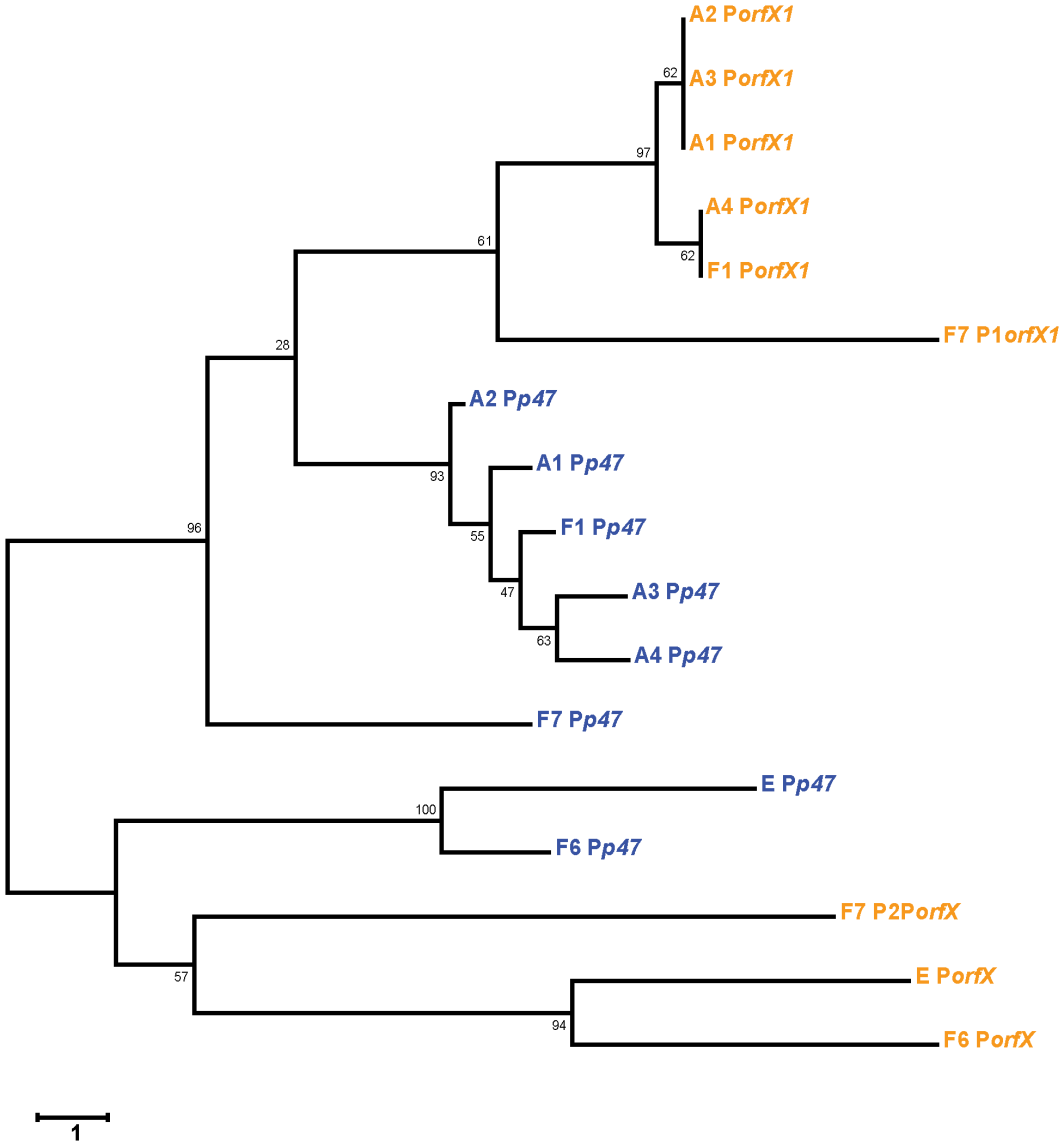
Phylogenetic analysis of the botulinum neurotoxin gene cluster promoters. The P*p47* and P*orfX1* promoter sequences presented in Fig. 2 (upstream of the *p47* and *orfX1* transcripts, respectively) were aligned and a phylogenetic tree was drawn to scale using the Neighbor-Joining method, with branch lengths in the same units as the evolutionary distances used to infer the phylogenetic tree. All positions containing gaps and missing data were eliminated from the dataset (complete deletion option). Phylogenetic analyses were conducted in MEGA4 software. Bootstrap values and number of base differences per site are shown. Note that the F7 P1*orfX1* promoter sequence is phylogenetically more related to its type A and F1 homologs, while the F7 P2*orfX1* promoter is more related to its type E and F6 homologues. This finding implies that the F7 *orfX1* transcript may be regulated by a BotR-like protein through its P1 promoter and by a type E or type F6-like regulator through its P2 promoter.

We performed a phylogenetic analysis of the P*p47* and P*orfX1* promoter sequences displayed in Figure 2A. We found that the nonproteolytic *C. baratii* F7 P1*orfX1* promoter is more closely related to the proteolytic *C. botulinum* P*orfX1* promoters in subtypes A1–A4 and F1, while the *C. baratii* F7 P2*orfX1* promoter is more closely related to the nonproteolytic *C. botulinum* and *C. butyricum* P*orfX1* promoters (Fig. 4). The *C. baratii* F7 P*p47* promoter was more closely related to P*p47* in proteolytic clostridia (Fig. 4).

### Identification and characterization of a TcdR-related, Uvia-like putative σ factor

**Figure 5:**
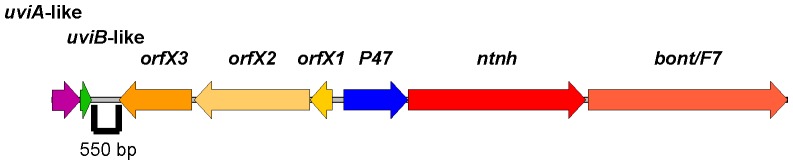
Arrangement of the *C. baratii* type F7 neurotoxin gene cluster and its associated *uviAB*-like operon. The type F7 toxin gene cluster arrangement with its upstream *uviAB*-like operon are shown.

Because no *botR* gene exists within the *C. baratii* F7 toxin gene cluster (Fig. 1) and because the cluster contained predicted −35 promoter elements identical to the sequence recognized by TcdR-related regulatory proteins (Fig. 2), we searched elsewhere for a *tcdR*-like regulatory gene. In the flanking regions of the F7 toxin gene cluster of *C. baratii* strain IBCA03-0045 we identified a putative operon of two open reading frames (ORFs) with an overlap of 20 bp that was located 550 bp upstream of *orfX3* and resembled in size and gene arrangement the *C. perfringens uviAB* operon (Fig. 5 and [27]). The larger, *uviA*-like (∼55% nucleic acid identity) ORF consisted of 555 bp, which is similar in length both to the *botR* gene (which ranges from 537 bp to 546 bp) in proteolytic *C. botulinum* strains and to genes that encode for members of the TcdR-related family of σ factors [12]. The smaller ORF consisted of 222 bp and was *uviB*-like (∼58% nucleic acid identity). Sequencing of the equivalent upstream region (∼1500 bp) of the F7 toxin gene cluster in six additional toxigenic *C. baratii* strains isolated from six different infant botulism patients found that all of them contained this *uviAB*-like operon (data not shown).

Conserved Domain [35] and CDART [36] queries on the NCBI website of the predicted translated amino acid sequence of the *C. baratii uviA*-like upstream ORF identified domains that matched the botulinum-toxin-associated regulatory protein BotR, as well as domains that matched sequences within the RNA polymerase σ^70^ subunit regions 2 and 4. The σ^70^ regions 2 and 4 recognize the −10 and −35 promoter elements, respectively [37]. Dupuy et al. reported that UviA is a TcdR-related σ factor that regulates the expression of bacteriocin BCN5 of *C. perfringens* [27]. The role of UviB is unknown [27].

We also identified similar *uviA*-like genes in *uviAB*-like operons in the genome sequences of nonproteolytic *C. botulinum* type E strain Alaska (GenBank accession number CP001078 region: 866476-867021), in nonproteolytic *C. botulinum* type B strain Eklund 17B (GenBank accession number CP001056 region: 887914-888459) and in nonproteolytic *C. botulinum* type F6 strain IBCA66-5436 (GenBank accession number JX847736). These *uviA*-like genes are not within close proximity to the botulinum toxin gene clusters (e.g., 303,631 bp downstream of *bont/E* in *C. botulinum* type E strain Alaska). The *uviA*-like genes of nonproteolytic *C. botulinum* types B, E and F6 were 95%–99% identical to each other and were ∼50% identical to the *uviA*-like gene in *C. baratii* type F7. However, we could not find a similar *uviA*-like gene in the draft genomes of the nonproteolytic neurotoxigeneic *C. butyricum* type E strains BL5262 and 5521 (GenBank accession numbers NZ_ACOM00000000 and NZ_ABDT00000000, respectively).

**Figure 6:**
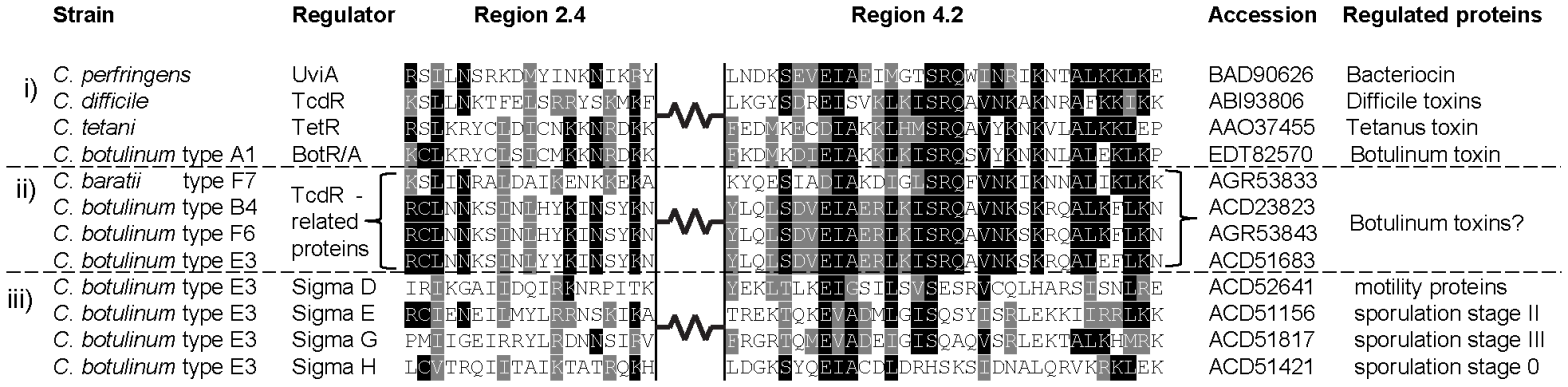
Alignment of the 2.4 and 4.2 regions of TcdR-related σ factors of selected toxigenic clostridia. Note the highly conserved Serine-Arginine-Glutamine (SRQ) motif in all 4.2 regions of the eight TcdR-related σ factors (i and ii). The alignment includes the 2.4 and 4.2 regions of: i) the TcdR-related σ factors UviA (*C. perfringens*), TcdR (*C. difficile*), TetR (*C. tetani*), BotR (*C. botulinum* type A) (adapted from [27]) and ii) the putative TcdR-related σ factors of *C. baratii* type F7, nonproteolytic *C. botulinum* type B4, *C. botulinum* type E3 and *C. botulinum* type F6. For comparison, (iii) four non-TcdR-related members of the σ^70^ family found in *C. botulinum* type E3 strain Alaska (σ^E^, σ^G^, σ^H^ and σ^D^) were also included in the alignment. BoxShade (http://www.ch.embnet.org/software/BOX_form.html) with a threshold of 0.5 was used for shading of identical amino acids (black) and similar amino acids (grey). Accession numbers are from GenBank. The zigzag sign represents the non-continuity of the 2.4 and 4.2 regions.

Members of the TcdR-related σ factors share the conserved region 4.2, which is the region that interacts with the −35 promoter element, while region 2.4 that interacts with the −10 promoter element is more variable [12], [27]. We aligned the four nonproteolytic *C. botulinum* and the nonproteolytic *C. baratii* UviA-like regions 2.4 and 4.2 predicted amino acid sequences with those of TcdR, UviA, TetR and BotR. Remarkably, all eight sequences shared the same conserved region 4.2 (Fig. 6 i and ii). All eight sequences also contained the highly conserved Serine-Arginine-Glutamine motif (SRQ) that is absent in non-TcdR-related alternative σ factors also belonging to the σ^70^ family ([12] and Fig. 6iii). As reported before, the amino acids sequence of region 2.4 varied among the TcdR-related σ factors [12].

### Nontoxigenic *C. baratii*

**Figure 7:**
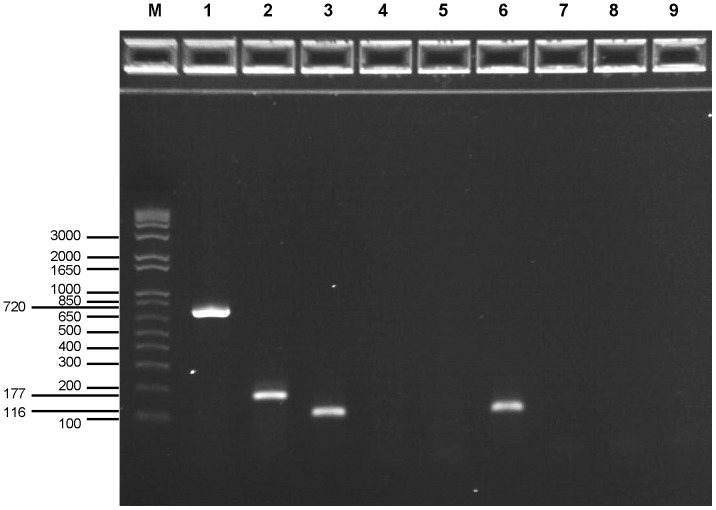
PCR amplification comparison of toxigenic and nontoxigenic *C. baratii* strains. Genomic DNA isolated from toxigenic (strain IBCA03-0045) (lanes 1–3) and nontoxigenic (strain IBCA08-0076) (lanes 4–6) *C. baratii* was used as templates. No template PCR (lanes 7–9) was used as a negative control. The PCR was performed with primer pairs specific for *bont/F7* (primers baratii58F and baratii60R, lanes 1, 4 and 7), the *uviA*-like gene (primers baratii1F and baratii2R, lanes 2, 5 and 8), and the 23S rRNA gene (primers baratii23sF1 and baratii23sR1, lanes 3, 6 and 9) of *C. baratii*. The toxigenic *C. baratii* strain was PCR-positive for both the *bont/F7* and the *uviA*-like genes (lanes 1 and 2), while the nontoxigenic strain was PCR-negative for both these genes (lanes 4 and 5). This finding suggests that the *C. baratii bont/F7* neurotoxin gene and the *uviA*-like gene are genetically linked. Note that both *C. baratii* strains were PCR-positive for the 23S rRNA gene that served as a positive control (lanes 3 and 6). M  =  Molecular weight markers (1 Kb Plus DNA Ladder, Invitrogen). The sizes (in base pairs) of the Molecular weight markers and the amplicons are presented at the left.

To determine whether the *C. baratii uviA*-like gene and *bont/F7* were genetically linked, DNA extracted from neurotoxigenic *C. baratii* strain IBCA03-0045 type F7 and from nontoxigenic *C. baratii* strain IBCA08-0076 were tested by PCR for the presence of both genes. As expected, the neurotoxigenic *C. baratii* strain was PCR-positive for both the *bont/F7* and the novel *uviA*-like genes. In contrast, the nontoxigenic *C. baratii* strain was PCR-negative for both genes (Fig. 7). Identical results were obtained for an additional six toxigenic and two nontoxigenic *C. baratii* strains (data not shown).

### UviA-like protein functions like a σ factor that enables RNA polymerase core enzyme binding to the F7 botulinum toxin gene cluster promoters

**Figure 8:**
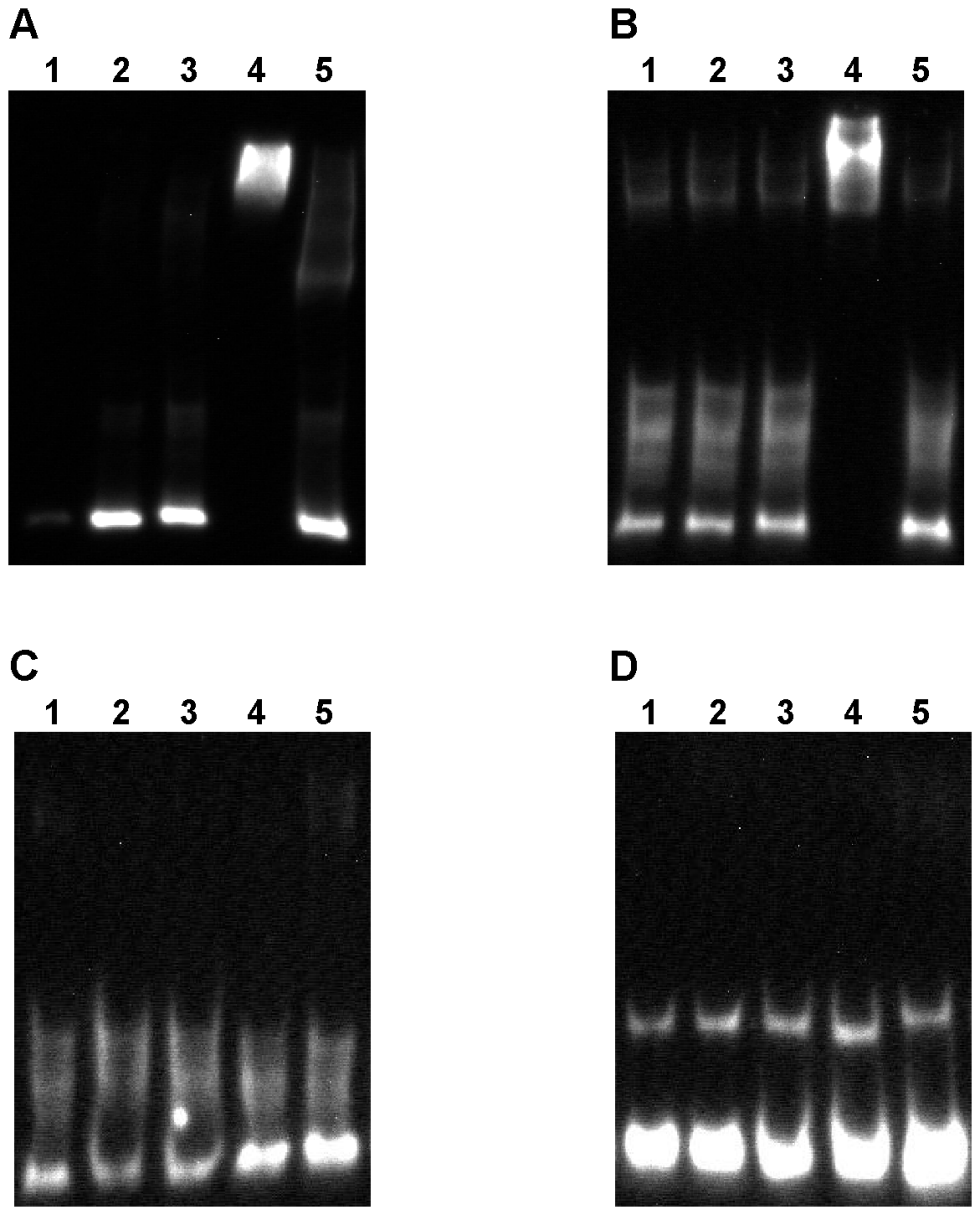
Gel mobility shift assays of the F7 botulinum gene cluster promoters with the UviA-like protein. Labeled DNA fragments containing (A) or lacking (C) the P*p47* and the P2*orfX1* promoters and containing (B) or lacking (D) the P1*orfX1* promoter were tested in gel mobility shift assays. The labeled DNA (lane 1) was incubated with RNA polymerase core (lane 2), UviA-like protein (lane 3), UviA-like protein preincubated with RNA polymerase core (lane 4) and UviA-like protein preincubated with RNA polymerase core and an excess of unlabeled DNA fragment (lane 5). Only the UviA-like-RNA polymerase core complex bound the botulinum toxin gene cluster promoters (lanes A4 and B4) and induced a shift in the labeled DNA migration. An excess of unlabeled DNA (lanes A5 and B5) or elimination of the promoter sequences (lanes C4 and D4) prevented the binding.

We overexpressed and purified the *C. baratii* type F7 UviA-like protein in order to study its association with the botulinum toxin gene cluster promoters in gel mobility shift assays. Fig. 8 displays the electrophoretic mobility of 250 bp DNA fragments that contained the overlapping P*p47* and P2*orfX1* promoters (Fig. 8A) or the P1*orfX1* promoter (Fig. 8B). RNA polymerase core enzyme and the purified UviA-like protein by themselves did not shift the migration of DNA fragments that contained the type F7 botulinum toxin gene cluster promoters (Fig. 8A and B, lanes 2 and 3). In contrast, following preincubation of the purified UviA-like protein and RNA polymerase core, a shift in migration was observed (Fig. 8A and B, lane 4), indicating that the UviA-like-RNA polymerase core complex bound to both promoter-containing DNA fragments. An excess of unlabeled homologous DNA fragments prevented the binding of the labeled fragments, indicating specific interactions (Fig. 8A and B, lane 5).

We repeated the gel mobility shift assays described above with similar DNA fragments that did not contain any promoter regions (Fig. 8C and D). In these DNA fragments the promoter sequences were replaced with a random nucleotide sequence. No shift in migration of either DNA fragment was observed, further establishing the specificity of the binding of the UviA-like-RNA polymerase core complex to the type F7 botulinum toxin gene cluster promoters.

## Discussion

Here we report the sequence and arrangement of the entire botulinum toxin gene cluster of nonproteolytic *C. baratii* type F7. Its *orfX* toxin gene cluster does not contain a *botR* gene, similarly to nonproteolytic *C. botulinum* type F6 [8], nonproteolytic *C. botulinum* type E and nonproteolytic *C. butyricum* type E toxin gene clusters (Fig. 1) [16]. We unexpectedly found that some of the *C. baratii* type F7 toxin gene cluster promoters are similar to the conserved BotR-recognized promoters (Figs. 2 and 4) [14]. We identified a gene coding for a putative UviA-like regulatory protein immediately upstream of the *C. baratii* type F7 toxin gene cluster that shared a similar DNA-binding-domain with BotR and the TcdR-related group 5 of the σ^70^ family (Fig. 5 and 6) [12], [27]. The purified UviA-like regulatory protein in complex with RNA polymerase core enzyme specifically recognized the *C. baratii* type F7 botulinum toxin gene cluster promoters (Fig. 8). Therefore, the UviA-like protein of *C. baratii* type F7 may serve as an alternative σ factor that recognizes the botulinum toxin gene cluster promoters of this organism.

In contrast to the types E and F6 *orfX* toxin gene clusters in nonproteolytic clostridia, the type B4 *ha* toxin gene cluster in the nonproteolytic strain Eklund 17B and the type G *ha* toxin gene cluster in the nonproteolytic strain ATCC 27322 both contain the *botR* gene [38]. Hence, the absence of the *botR* regulatory gene within the toxin gene cluster may be a general characteristic of the *orfX* toxin gene clusters that reside in nonproteolytic neurotoxigenic clostridia. If this generalization is correct, then regulation of *bont* expression in *orfX* toxin gene clusters may differ between the nonproteolytic and the proteolytic neurotoxigenic clostridia.

However, the −35 promoter element TTTACA that is associated with σ factor recognition and transcription of the polycistronic mRNA that codes for BoNT is conserved in all analyzed *bont* toxin gene clusters (Fig. 2i and [17]), as well as in other clostridial toxin gene promoters [22]. This promoter sequence conservation suggests that a similar transcription regulator participates in the expression of all *bont* toxin gene clusters (and possibly of other clostridial toxin genes), regardless of the presence or absence of the *botR* regulatory gene.

The nonproteolytic *C. baratii* type F7 P*p47* and P1*orfX1* toxin gene cluster promoter sequences differed from their *orfX* toxin gene cluster promoters in nonproteolytic *C. botulinum* types E and F6 and were more homologous to the conserved promoters in proteolytic *C. botulinum* strains that are recognized by BotR (Fig. 2 and Fig. 4). This homology implies that the *C. baratii* P*p47* and P1*orfX1* promoters might be recognized by a BotR-like regulatory protein belonging to the TcdR-related group 5 subfamily of σ factors.

Interestingly, the predicted P*orfX1* −35 promoter elements in the nonproteolytic type E and F6 strains (TTGAAA and TTGTAA, respectively) differed from the highly conserved −35 element (TTTACA) recognized by the TcdR-related group 5 subfamily of σ factors (Fig. 2). Additionally, the P*orfX1* −10 promoter element in nonproteolytic type E and F6 strains was identical to the primary σ factor (*E. coli* σ^70^ and *B. subtilis* σ^A^) consensus recognition sequence TATAAT. Promoters that contain the −35 TTGAAA and −10 TATAAT motifs are known to be primary σ^A^ -dependent promoters in clostridia [33]. The *C. baratii* P2*orfX1* promoter sequence is more similar to the P*orfX1* promoters in nonproteolytic type E and F6 strains than to the *C. baratii* P1*orfX1* and the P*orfX1* promoters in the proteolytic strains (Fig. 4).

Although the P*p47* −35 promoter motif in nonproteolytic type E and F6 strains was identical to the conserved −35 motif recognized by TcdR-related σ factors, their −10 promoter motif (TATATT) was highly homologous to the σ^A^ consensus recognition sequence (TATAAT) (Fig. 2). It is intriguing to speculate that the *orfX1* transcript, and perhaps even the *p47* transcript in nonproteolytic type E and F6 strains, are transcribed by an RNA polymerase that utilizes the primary σ factor (σ^A^), rather than the alternative σ factor (BotR) that is used by all proteolytic *C. botulinum* [14].

In *C. baratii* type F7 we identified a putative regulatory gene immediately upstream of the toxin gene cluster, as also occurs with the toxin gene clusters of *C. botulinum* types C and D [20], [39]. But unlike *C. botulinum* types C and D, the *C. baratii* upstream putative regulatory gene is part of an operon with a similar size, structure and sequence to the *uviAB* operon of *C. perfringens* that codes for UviA and UviB [27]. Like BotR, UviA is a member of the TcdR-related group 5 of the σ^70^ family and regulates the expression of a toxin (bacteriocin BCN5), while the role of UviB is unknown [25], [27]. The presence of similar regulatory genes that are involved in the regulation of unrelated toxins in different clostridium species may represent horizontal gene transfer and subsequent independent evolution [40]. Predicted amino acid sequence analysis found that the UviA-like protein of *C. baraii* type F7 is also a member of the σ^70^ family. Moreover, the UviA-like protein of *C. baraii* type F7 contained the same conserved region 4.2 of the TcdR-related group 5 subfamily of σ factors that recognizes the conserved −35 promoter element TTTACA (Fig. 6). The highly conserved and unique Serine-Arginine-Glutamine (SRQ) motif in region 4.2 of the TcdR-related group 5 subfamily of σ factors may be a useful marker for recognizing members of this group.

However, the predicted amino acid sequence of region 2.4 of the UviA-like protein of *C. baratii* type F7 is quite different from the amino acid sequence of region 2.4 of the other TcdR-related σ factors (Fig. 6). Dupuy et al. reported that region 2.4 of the TcdR-related σ factors, the region responsible for the recognition of the −10 promoter element, is not conserved. This observation explains the sequence variability of the −10 promoter elements of the promoters that are recognized by TcdR-related σ factors [12].

We used gel mobility shift assays to show that the UviA-like protein of *C. baratii* type F7, in association with RNA polymerase core, binds specifically a DNA fragment that contains both the P*p47* and P2*orfX1* promoters and to a DNA fragment that contains the P1*orfX1* promoter. The binding of UviA-like protein in complex with RNA polymerase core (Fig. 8A and B lane 4), but not by itself (Fig. 8A and B lane 3), to the DNA fragments that contained the promoters demonstrates the recognition of the promoter sequences by the UviA-like protein in playing the role of a σ factor.

The *uviA*-like putative regulatory gene of *C. baratii* could not be detected by PCR in nontoxigenic strains that lack *bont/F7* (Fig. 7). This finding indicates that the two genes are genetically linked and suggests that they may also be functionally related. However, gene sequence variability may also have contributed to the negative PCR results. Genomic sequence comparison of toxigenic and nontoxigenic *C. baratii* strains is needed to confirm the absence of the *uviA*-like gene in nontoxigenic *C. baratii* strains.

We located similar *uviAB*-like operons that encode putative TcdR-like σ factors in other nonproteolytic neurotoxigenic clostridia that, like nonproteolytic *C. baratii*, lack the *botR* gene (e.g., *C. botulinum* type E and *C. botulinum* type F6). However, the *uviAB*-like operon resides in close proximity to the *bont* toxin gene cluster only in the genome of *C. baratii*. Also, *C. baratii* was the only nonproteolytic neurotoxigenic *Clostridium* that contained *orfX* toxin gene cluster promoters similar to the toxin gene cluster promoters in proteolytic neurotoxigenic clostridia (Figs. 2 and 4). Therefore, it appears that *C. baratii* type F7 may have a unique molecular mechanism that controls the expression of its botulinum toxin gene cluster. If so, this unique mechanism may help explain the special clinical features observed in type F7 infant botulism patients.

## Supporting Information

Table S1 **Primers used for amplification, sequencing and mapping of various genes.**
(DOCX)
