# Supplementary material for: Arrangement of the Clostridium baratii F7 Toxin Gene Cluster with Identification of a σ Factor That Recognizes the Botulinum Toxin Gene Cluster Promoters
Source: PLoS One. 2014 May 22;9(5):e97983. doi: 10.1371/journal.pone.0097983 (PMC4031146; doi:10.1371/journal.pone.0097983)
Supplement: Table S1 — Primers used for amplification, sequencing and mapping of various genes. (DOCX) [file pone.0097983.s001.docx]

**Table S1 - Primers used for amplification, sequencing and mapping of various genes.**

| Primer^1,2^ | Use^3^ | Sequence | Accession No. | Location^4^ |
| --- | --- | --- | --- | --- |
| baratii1F | A | TGATTTGGAGGATACTTTAAGGAG | JX847735 | 547-570 |
| baratii2R | A | TGCATCTAAAGCTCTATTTATGAGTGA | JX847735 | 698-724 |
| baratii3F | A | GCAATGTCACAAGGTATTTGGA | JX847735 | 1038-1059 |
| baratii4R | A | TTTTTCTCTTTGCTCCTGTCTT | JX847735 | 1118-1139 |
| baratii5F | A, S | GAAAGGGCTTTAAATAAAACAAAAA | JX847735 | 1-25 |
| baratii6F | S | AAAGGGGGAAAGCTTTATGA | JX847735 | 463-482 |
| baratii7R | S | AAAGCCTTTTTATCTCCTTTCTGA | JX847735 | 508-531 |
| baratii8R | S | CAAACTGCCTTGACAGTCCT | JX847735 | 931-950 |
| baratii9F | S | AGGACTGTCAAGGCAGTTTG | JX847735 | 931-950 |
| baratii10R | S | CAACAAAAAGGCATAATCATTCA | JX847735 | 1315-1337 |
| baratii11F | A, S | ATGCCTTTTTGTTGAAAATCA | JX847735 | 1324-1344 |
| baratii12F | S | TTGATTTTGCATAGCTCTTTTTG | JX847735 | 1393-1415 |
| baratii13R | A, S | AAAATTCTGTTTCTGAAATTACATGG | JX847735 | 1872-1897 |
| baratii14F | S | GCTTAAAGCAGAAGATAAGAATGC | JX847735 | 2043-2066 |
| baratii15R | S | TCAAAGTAATGAAAGTGGTGGA | JX847735 | 2382-2403 |
| baratii16F | S | TGGAATCAAATTTTTGTTGCTG | JX847735 | 2531-2552 |
| baratii17R | S | TAATGCTGAAGATCCGGCTA | JX847735 | 2876-2895 |
| baratii18F | S | GGAATTTTAAGTCTAATATTACCACCA | JX847735 | 3064-3090 |
| baratii19R | S | GTGCTGTACAATGGCCAGAT | JX847735 | 3380-3399 |
| baratii20F | S | TTGTCCAAATGGTTTTGCTG | JX847735 | 3566-3585 |
| baratii21R | S | TGGTAACAGAAGAAGCTTGGAA | JX847735 | 3894-3915 |
| baratii22F | S | TGTTGCCAAGTTGCATCAAT | JX847735 | 4035-4054 |
| baratii23R | S | TTTTTGCAGCAATGGCTATG | JX847735 | 4364-4383 |
| baratii24F | S | TTGACCATTATTTAATCCAGTAGGG | JX847735 | 4694-4718 |
| baratii25F | A, S | TTGCGTAGGCTTTAGCCATT | JX847735 | 4457-4476 |
| baratii26F | S | TGTTCGAATTTTGCTATATTCTCATT | JX847735 | 4527-4552 |
| baratii27R | S | AGTTTTTAGAAAATGGTTTAATGAGAA | JX847735 | 4545-4571 |
| baratii28R | S | GGTCCTTGGCAAATGACTACA | JX847735 | 4868-4888 |
| baratii29R | S | AGGATGGGATATTGAAAGTGC | JX847735 | 5001-5021 |
| baratii30F | S | AGATTTTCTTCAGTCTTGGTTTGT | JX847735 | 5031-5054 |
| baratii31F | S | CCATCTGAATTAGGTCTAATGGTCA | JX847735 | 5214-5238 |
| baratii32R | A, S | CGGAGGGAAAGTCTTCAAATC | JX847735 | 5397-5417 |
| baratii33R | S | GAGCCATTTGTATATTATGATTGGAA | JX847735 | 5496-5521 |
| baratii34F | S | CCTATTTTCCAATTTCATGTCTTTT | JX847735 | 5682-5706 |
| baratii35R | S | GGAGGTGAAAACATGAATGAAA | JX847735 | 5960-5981 |
| baratii36R | S | CCATTTAAATTAGTGGTTGCATTTC | JX847735 | 6470-6494 |
| baratii37F | S | GAGTGGATGAATCCTAAACAATTT | JX847735 | 6742-6765 |
| baratii38R | S | TGCTGTAGTATCGGATGTACTTATGAC | JX847735 | 6994-7020 |
| baratii39F | S | CAGGATATGAGATAGTTTACGCTGAT | JX847735 | 7163-7188 |
| baratii40R | S | ATATTTTCACCTTCTTTAATTTGCTTT | JX847735 | 7486-7512 |
| baratii41F | S | GATCAAAAGTCTGATGGAGGAA | JX847735 | 7678-7699 |
| baratii42R | S | TGGTTGAAACCATACTTCACACA | JX847735 | 8006-8028 |
| baratii43F | A, S | AAATTTTTGAACACTAATCCTTACTGG | JX847735 | 8233-8259 |
| baratii44R | A, S | TTTGCCCATTTACAAAACCA | JX847735 | 8538-8557 |
| baratii45F | A, S | GCAAGCCCAAATGACAAATAA | JX847735 | 8922-8942 |
| baratii46F | S | AGTACCATGGATTGGCAAAG | JX847735 | 9171-9190 |
| baratii47R | A, S | TGTGTCCACCACTGATCTAAGAA | JX847735 | 9409-9431 |
| baratii48F | S | TGGGGATGGATCAGGTAAAA | JX847735 | 9960-9979 |
| baratii49R | S | TTACCTGATCCATCCCCAAT | JX847735 | 9958-9977 |
| baratii50F | S | AACGATGTTCTTGTTGCAAAT | JX847735 | 10348-10368 |
| baratii50R | S | TCATTTGCAACAAGAACATCG | JX847735 | 10350-10370 |
| baratii51F | S | AAAAATATGTTCAAAAATGTGATGAAG | JX847735 | 10712-10738 |
| baratii52F^1^ | A, S | ATAGTAGATAATAAGAGTAGTGCAAAT | JX847735 | 10807-10833 |
| baratii53R | S | ATGGCATTTTCATGTACAAAATAG | JX847735 | 11067-11090 |
| baratii54F^1^ | A, S | GTAATCCTGCAGGACAAG | JX847735 | 11313-11330 |
| baratii55R^1^ | S | CTTGTCCTGCAGGATTAC | JX847735 | 11313-11330 |
| baratii56R | S | CGGCAGTATGGTCATTTCCT | JX847735 | 11567-11387 |
| baratii57R^1^ | A, S | ATAATATTTAAATCTTGACCTCCAAAG | JX847735 | 11797-11823 |
| baratii58F^1^ | A, S | TCGCCTAAAGAAATTGACG | JX847735 | 12389-12407 |
| baratii59R^1^ | A, S | CGTCAATTTCTTTAGGCGA | JX847735 | 12389-12407 |
| baratii60R^1^ | A, S | GCCAATTTGATACTATCCAACTA | JX847735 | 13087-13109 |
| baratii61F^1^ | A, S | TAGTTGGATAGTATCAAATTGGC | JX847735 | 13087-13109 |
| baratii62F^1^ | S | AACATTAGGAACTAGTAGCGT | JX847735 | 13447-13467 |
| baratii63R^1^ | A, S | TTCCAGTTGTATCTTGCAAT | JX847735 | 13927-13946 |
| baratii64F^1^ | A, S | ATTGCAAGATACAACTGGAA | JX847735 | 13927-13946 |
| baratii65R^1^ | S | ACTATAAATCCCTCTTTGTCTATTA | JX847735 | 14362-14386 |
| baratii66F^1^ | S | TAATAGACAAAGAGGGATTTATAGT | JX847735 | 14362-14386 |
| baratii67R^1^ | S | TTATTCTTGCCATCCATGT | JX847735 | 14803-14821 |
| baratii68R | A, S | TGGTGGGTCTGTTTATGCTG | JX847735 | 15110-15129 |
| baratii23sF1 | A, S | TGGATCAGAACCTTAGTCATTATCAA | JX847735 | 1-26 |
| baratii23sR1 | A, S | GGTTGCGGACAGAACATAAAGAG | JX847735 | 94-116 |
| baratii23sF2 | S | CATTATCAATTTCATCTACAGGACT | JX847735 | 18-42 |
| baratii23sR2 | S | AATAATCGAAGATAACTGGAAAGT | JX847735 | 61-84 |
| B27F^2^ | A, S | AGAGTTTGATCCTGGCTCAG | AB567726 | 1-20 |
| U1492R^2^ | A, S | GGTTACCTTGTTACGACTT | AB567726 | 1458-1476 |
| 928F^2^ | S | TAAAACTYAAAKGAATTGACGGG | AB567726 | 871-893 |
| 336R^2^ | S | ACTGCTGCSYCCCGTAGGAGTCT | AB567726 | 328-350 |
| 1100F^2^ | S | YAACGAGCGCAACCC | AB567726 | 1066-1080 |
| 1100R^2^ | S | GGGTTGCGCTCGTTG | AB567726 | 1066-1080 |
| 337F^2^ | S | GACTCCTACGGGAGGCWGCAG | AB567726 | 329-349 |
| 907R^2^ | S | CCGTCAATTCCTTTRAGTTT | AB567726 | 873-892 |
| 785F^2^ | S | GGATTAGATACCCTGGTA | AB567726 | 752-769 |
| 805R^2^ | S | GACTACCAGGGTATCTAATC | AB567726 | 753-772 |
| 533F^2^ | S | GTGCCAGCMGCCGCGGTAA | AB567726 | 482-500 |
| 518R^2^ | S | GTATTACCGCGGCTGCTGG | AB567726 | 485-503 |
| F7p47-GSP1 | RACE | TCTTTCACTAATACCAGAACCCATA | JX847735 | 6960-6984 |
| F7p47-GSP2 | RACE | TTCATCTTCTTCTTGTAGTTTTCCA | JX847735 | 6624-6648 |
| F7p47-GSP3 | RACE | CCATTTAAATTAGTGGTTGCATTTC | JX847735 | 6470-6494 |
| F7p47-seq | RACE | TTTCCCAACCTTCAAAGTTCA | JX847735 | 6380-6400 |
| F7orfX-GSP1 | RACE | AATGCCATGATTTCCAAGTACC | JX847735 | 5365-5386 |
| F7orfX-GSP2 | RACE | AGATTTTCTTCAGTCTTGGTTTGT | JX847735 | 5031-5054 |
| F7orfX-GSP3 | RACE | AAACCTATTTTCCAATTTCATGTC | JX847735 | 5679-5702 |
| F7orfX-seq | RACE | AATTTTGAAATATTGGTAATTAGGAC | JX847735 | 5856-5881 |
| F6p47-GSP1 | RACE | CAGATCCCATATTACTCGAAAG | HQ441176 | 5660-5681 |
| F6p47-GSP2 | RACE | CCCTCGCTATTATCAGTTGGA | HQ441176 | 5494-5514 |
| F6p47-GSP3 | RACE | TTTCTTCATCCATTTTTCCA | HQ441176 | 5335-5354 |
| F6p47-seq | RACE | CCTCCATTAATAATTTCCCAAG | HQ441176 | 5103-5124 |
| F6orfX-GSP1 | RACE | TCCATGATTTCCAAGTTCCA | HQ441176 | 4057-4076 |
| F6orfX-GSP2 | RACE | TCCTCCCTTATGAAATTTGT | HQ441176 | 4229-4248 |
| F6orfX-GSP3 | RACE | GCAACCAAAGAGATTACACCATT | HQ441176 | 4462-4484 |
| F6orfX-seq | RACE | CTTCTTCTAGGATTCCATTTTTAGTT | HQ441176 | 4304-4329 |
| Ep47-GSP1 | RACE | GCTGCTACTTTAATCCCATACCA | AM695752 | 5169-5191 |
| Ep47-GSP2 | RACE | CCCATATTGCTTGAAAGTTTAGGT | AM695752 | 5063-5086 |
| Ep47-GSP3 | RACE | CATCATTATTATCAGTTGGAGAA | AM695752 | 4901-4923 |
| Ep47-seq | RACE | CCACTAAGATCTGAAACTATGACC | AM695752 | 4724-4747 |
| EorfX-GSP1 | RACE | TGGACCTTCACCTTCATCTG | AM695752 | 3442-3461 |
| EorfX-GSP2 | RACE | TCCAAGATTTCCAATTACCA | AM695752 | 3468-3487 |
| EorfX-GSP3 | RACE | TCCCTTTTTAAAGCAACCAAA | AM695752 | 3861-3881 |
| EorfX-seq | RACE | TGCAATTCTGTTTTACTTTTTCCA | AM695752 | 3988-4011 |

^1^Primers used for sequencing of the *bont/F7* gene were taken from reference [7].

^2^Primers used for amplification and sequencing of the *C. baratii* 16S rRNA gene were taken from: <http://en.wikipedia.org/wiki/16S_ribosomal_RNA>

^3^Primers usage: A = primer used for amplification; S = primer used for sequencing;

RACE = Primer used for promoter mapping with 5’ RACE analysis.

^4^The location of the primer sequence reported under the corresponding GenBank accession number.
